# A Hidden Airway Injury: Delayed Diagnosis of Tracheal Disruption After Penetrating Neck Trauma

**DOI:** 10.7759/cureus.113959

**Published:** 2026-08-04

**Authors:** Kaelan S Patel, Nimr Assaf, Angelo Federico, John Hilu

**Affiliations:** 1 Neurology, Corewell Health, Dearborn, USA; 2 Gastroenterology, Corewell Health, Dearborn, USA; 3 General Surgery, Corewell Health, Dearborn, USA; 4 Cardiothoracic Surgery, Corewell Health, Dearborn, USA

**Keywords:** cardiothoracic and vascular surgery, cutaneous emphysema, motor vehicle collision, penetrating neck injury, pneumomediastinum, tracheobronchial injury

## Abstract

Tracheobronchial injury is a rare but potentially life-threatening complication of both blunt and penetrating chest trauma. Early recognition is critical, as a delayed diagnosis is associated with increased morbidity and mortality. This case report highlights a rare and potentially fatal tracheobronchial injury encountered following a motor vehicle collision (MVC). The patient sustained penetrating thoracic trauma secondary to guardrail intrusion, resulting in an injury to the carina with associated respiratory compromise requiring emergent chest tube placement. During operative exploration, disruption of the carinal region was identified and subsequently managed with definitive surgical repair. The patient ultimately survived and had an uncomplicated postoperative recovery. This case highlights the importance of maintaining a high index of suspicion for traumatic airway injuries, as early recognition and timely operative intervention are critical to optimizing outcomes.

## Introduction

Traumatic injuries to the tracheobronchial tree are rare but potentially fatal complications of thoracic trauma, with recent literature continuing to emphasize the need for rapid recognition and individualized management [1,2]. Although uncommon, these injuries carry substantial morbidity and mortality because disruption of the central airway can lead to severe air leak, respiratory failure, and associated thoracic and vascular injuries [1,3]. The involvement of the carina is particularly rare, yet prompt diagnosis is crucial given the location at the airway bifurcation and the difficulty of obtaining access in unstable trauma patients [1,4].

Motor vehicle collisions (MVCs) remain a major mechanism of severe thoracic trauma. Most tracheobronchial injuries occur after blunt force, while penetrating airway injuries from foreign object intrusion are far less common [2,4,5]. Contemporary reports and reviews continue to note that diagnosis is frequently delayed because symptoms may be nonspecific and early radiographic findings may underestimate injury severity [1,2,6]. Findings such as a persistent pneumothorax or air leak after chest tube placement, failure of complete lung re-expansion, the presence of subcutaneous emphysema, and the development of respiratory distress should heighten suspicion for major airway disruption [2,5,6].

Current evidence supports early bronchoscopy and multidisciplinary evaluation to define the extent of injury and guide treatment [1,2,6]. While selected small stable tears may be managed nonoperatively in carefully chosen patients, larger defects, unstable airway injuries, or injuries associated with ongoing air leak or compromised ventilation generally require operative repair [1,3,6]. We present a rare case of traumatic carinal disruption caused by guardrail penetration during an MVC managed with emergent chest tube placement and definitive surgical repair. This report highlights the importance of maintaining a high index of suspicion for tracheobronchial injury in complex thoracic trauma and the value of prompt operative intervention in achieving a favorable outcome [1,2,6].

Similar cases have been reported in the literature, in which patients with traumatic cervical tracheal injuries initially presented with relatively stable vital signs and minimal respiratory symptoms before developing progressive subcutaneous emphysema, pneumomediastinum, and respiratory compromise, resulting in delayed recognition of the underlying airway injury [7,8]. Bronchoscopic guidance was critical for confirming the diagnosis, securing the airway, and facilitating definitive operative repair in these cases, consistent with the management and outcome observed in our patient [2,4,6-8].

## Case presentation

A 23-year-old healthy male presented as a trauma activation after being struck by his own vehicle during an MVC. The patient reported that his car had entered a ditch, and while he was standing in front of it, another vehicle traveling at approximately 60 miles per hour struck his vehicle. This collision caused his car to strike him and propel him approximately 15 feet into a guardrail. No loss of consciousness was noted at the time of the incident.

**Figure 1:**
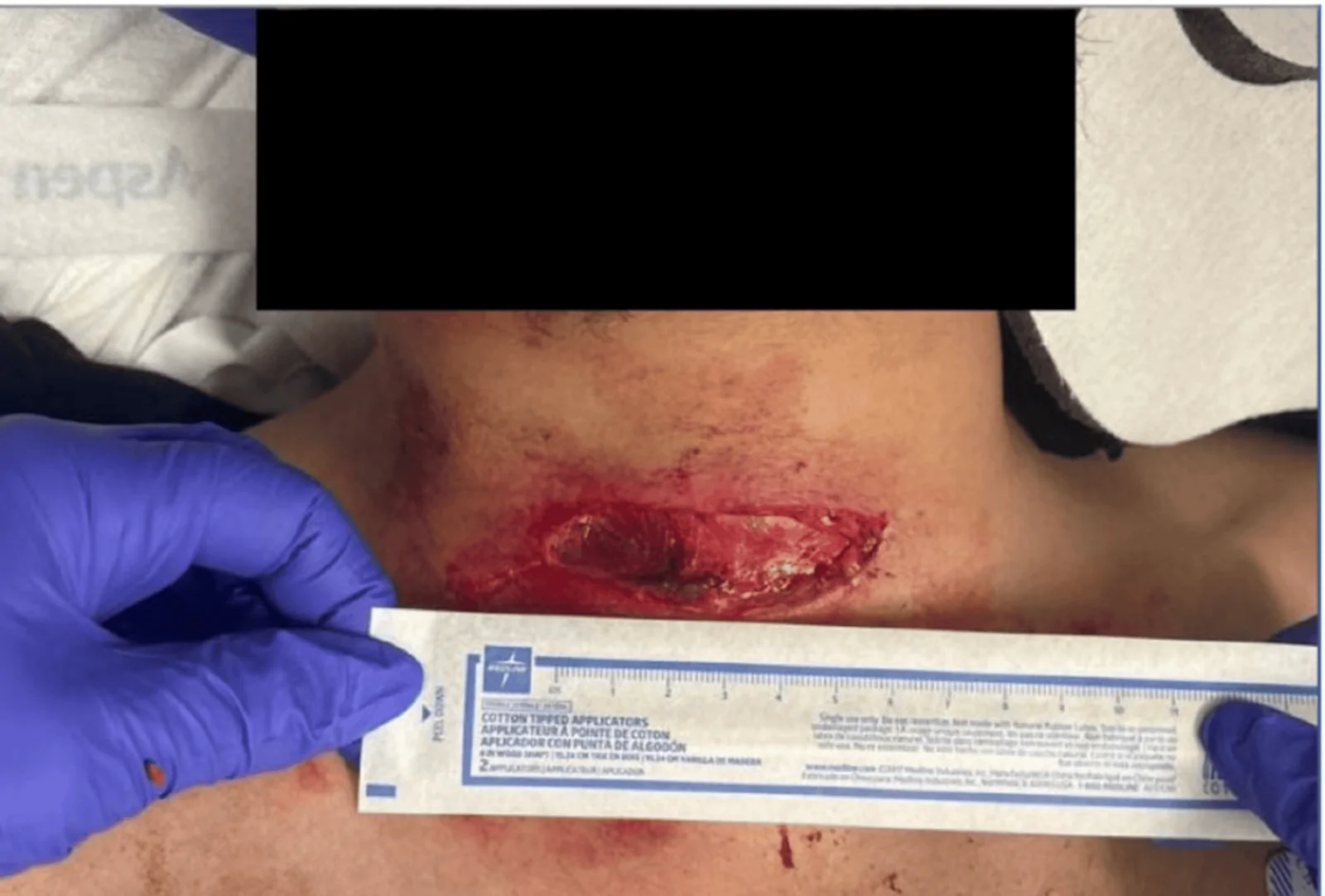
The patient’s transverse neck laceration, measuring 6 x 2 cm, and extending through the platysma.

On arrival, the patient was alert and oriented with a Glasgow Coma Scale score of 15. Vital signs were notable for blood pressure of 137/80 mmHg, heart rate of 117 beats/min, respiratory rate of 20 breaths/min, temperature of 37.1°C, and oxygen saturation of 100% on room air. Physical examination demonstrated a 6 × 2 cm transverse anterior neck laceration extending through the platysma with exposed musculature (Figure 1). His voice was noted to be high-pitched and hypernasal, although he denied subjective voice changes, dyspnea, or dysphagia. There was superior manubrial ecchymosis and posterior midline cervical tenderness, but no palpable cervical subcutaneous emphysema at initial evaluation. Breath sounds were equal bilaterally, and the remainder of the secondary survey was unremarkable. He had no significant past medical or surgical history, no known allergies, and denied tobacco, alcohol, or illicit drug usage.

**Figure 2:**
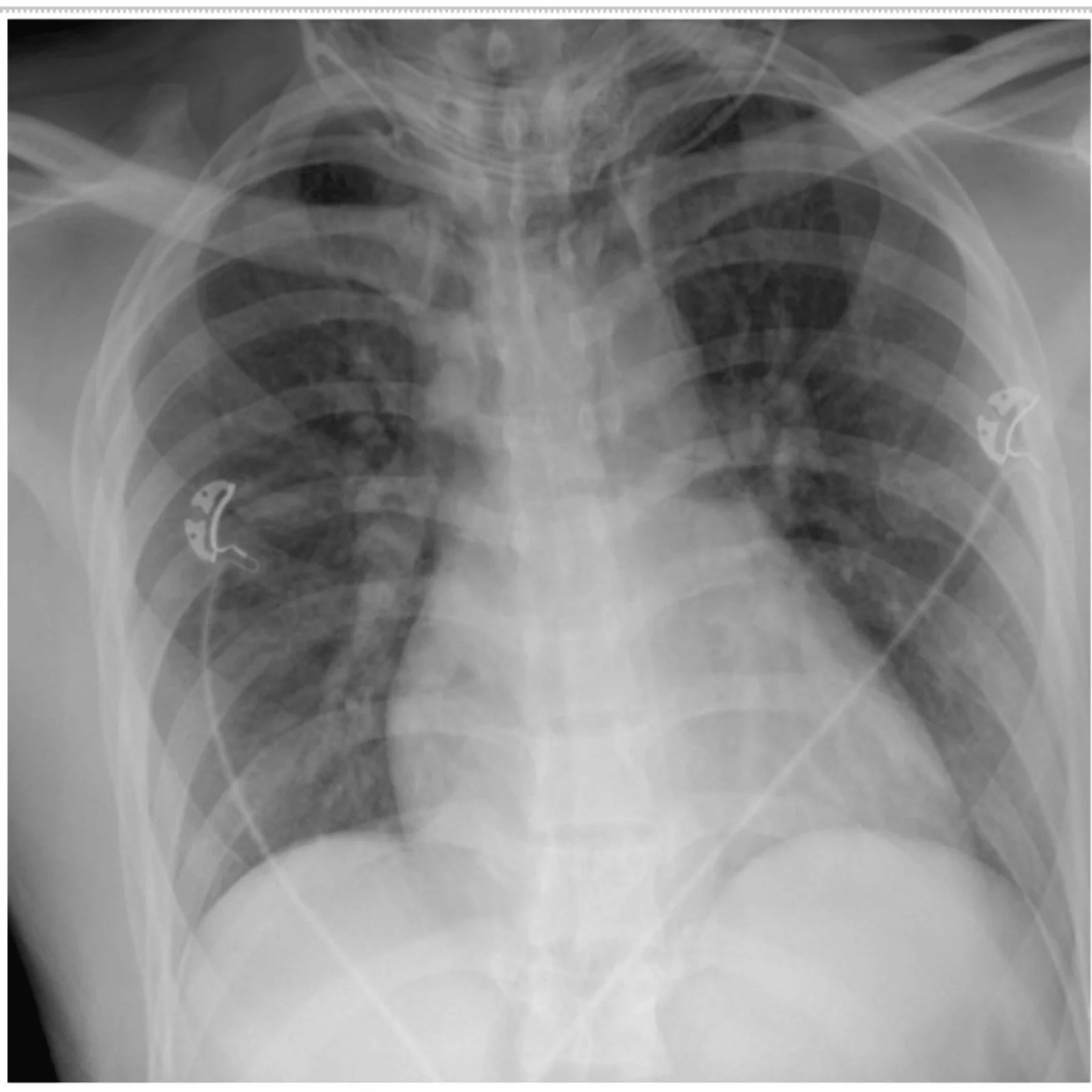
Chest X-ray with no acute cardiopulmonary process. Airway is midline. Cardiac silhouette within normal limits. No sizeable pleural effusion or pneumothorax. Cervical collar in place.

Initial chest radiography showed no acute intrathoracic abnormality (Figure 2). Intravenous cefazolin was administered for prophylaxis, given the presence of a penetrating neck injury. Shortly thereafter, the patient developed projectile emesis, facial swelling, and progressive respiratory distress, raising concern for an allergic reaction. He was subsequently treated with corticosteroids, diphenhydramine, and famotidine, but his symptoms continued to worsen. Given these symptoms, he was unable to tolerate lying supine to complete computed tomography (CT) imaging.

Approximately 75 minutes after arrival, repeat examination demonstrated marked bilateral cheek swelling, extensive subcutaneous emphysema involving the face and neck, and persistent tripoding. Family members at bedside reported a significant change in his phonation as well. Despite the presence of bilateral breath sounds and an oxygen saturation of 100%, concern for impending airway compromise prompted endotracheal intubation using video laryngoscopy. Mild airway edema and a small amount of blood at the vocal cords were noted while a 6.5-mm cuffed endotracheal tube was placed.

**Figure 3:**
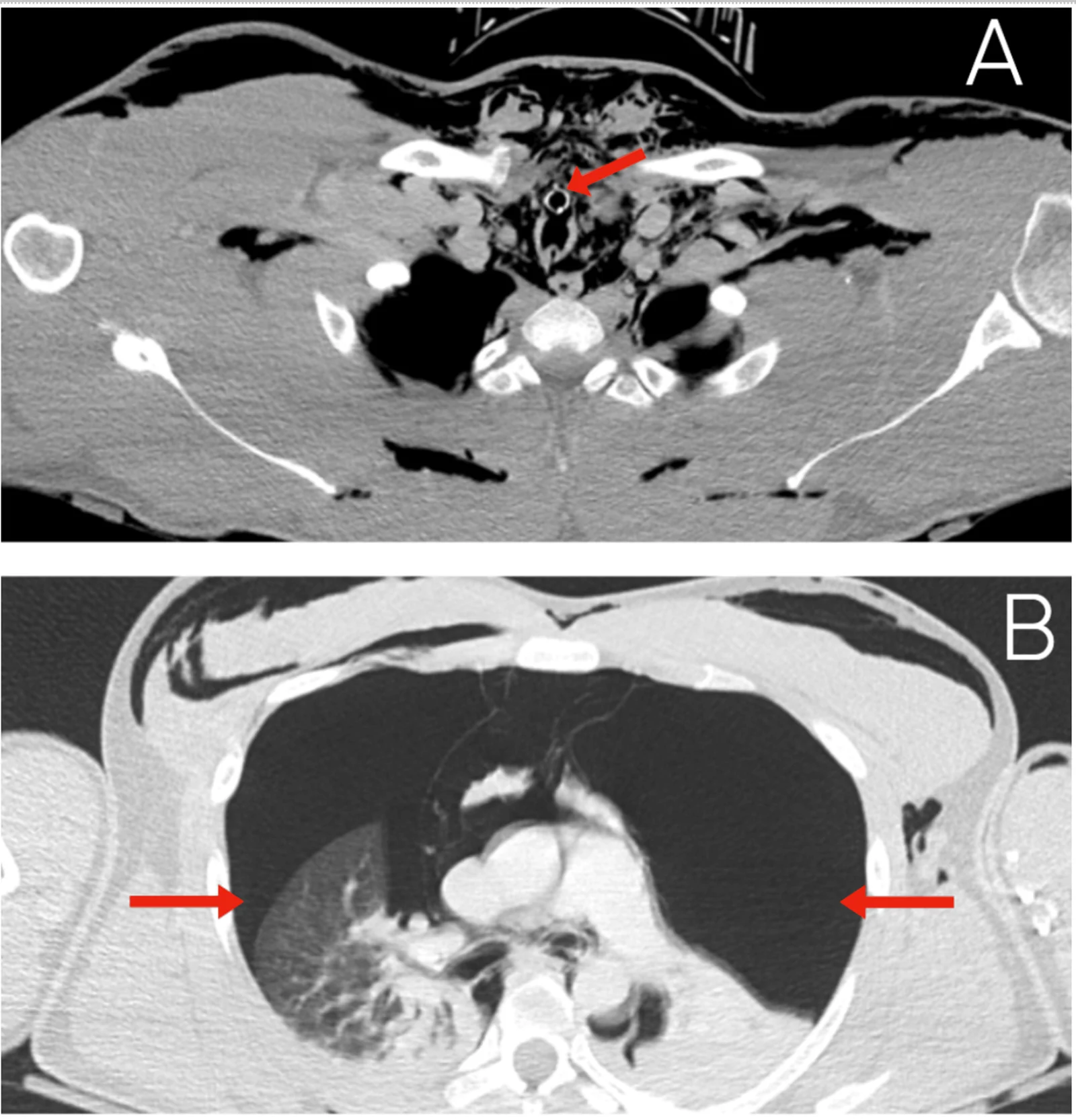
CT demonstrating traumatic airway injury and associated thoracic complications. A: CT of the neck showing rupture of the anterior tracheal wall with the endotracheal tube extending into the cervical soft tissues at the level of the superior border of the manubrium (red arrow); B: CT of the chest demonstrating large bilateral pneumothoraces with complete collapse of the left lung and partial collapse of the right lung (red arrows), along with extensive pneumomediastinum.

During subsequent CT imaging, peak inspiratory pressures rose significantly, and oxygen saturation decreased to 87%-92%. CT demonstrated large bilateral pneumothoraces, pneumomediastinum, and extensive subcutaneous emphysema extending throughout the chest and abdominal wall. Given the presence of bilateral pneumothoraces, bilateral tube thoracostomies were performed. On the initial finger sweep, a large release of air was noted bilaterally. On further review of the images, it was noted that the endotracheal tube had traversed a tracheal defect, with the tube tip positioned anterior to the distal tracheal lumen, as well as extensive pneumomediastinum (Figure 3). The patient remained difficult to ventilate with oxygen saturation in the low 90s. An emergent flexible bronchoscopy was performed in the emergency department, which allowed withdrawal and advancement of the endotracheal tube under direct visualization into the distal trachea beyond the site of injury. Immediately following this adjustment of the endotracheal tube, the patient’s oxygenation and ventilation status improved.

**Figure 4:**
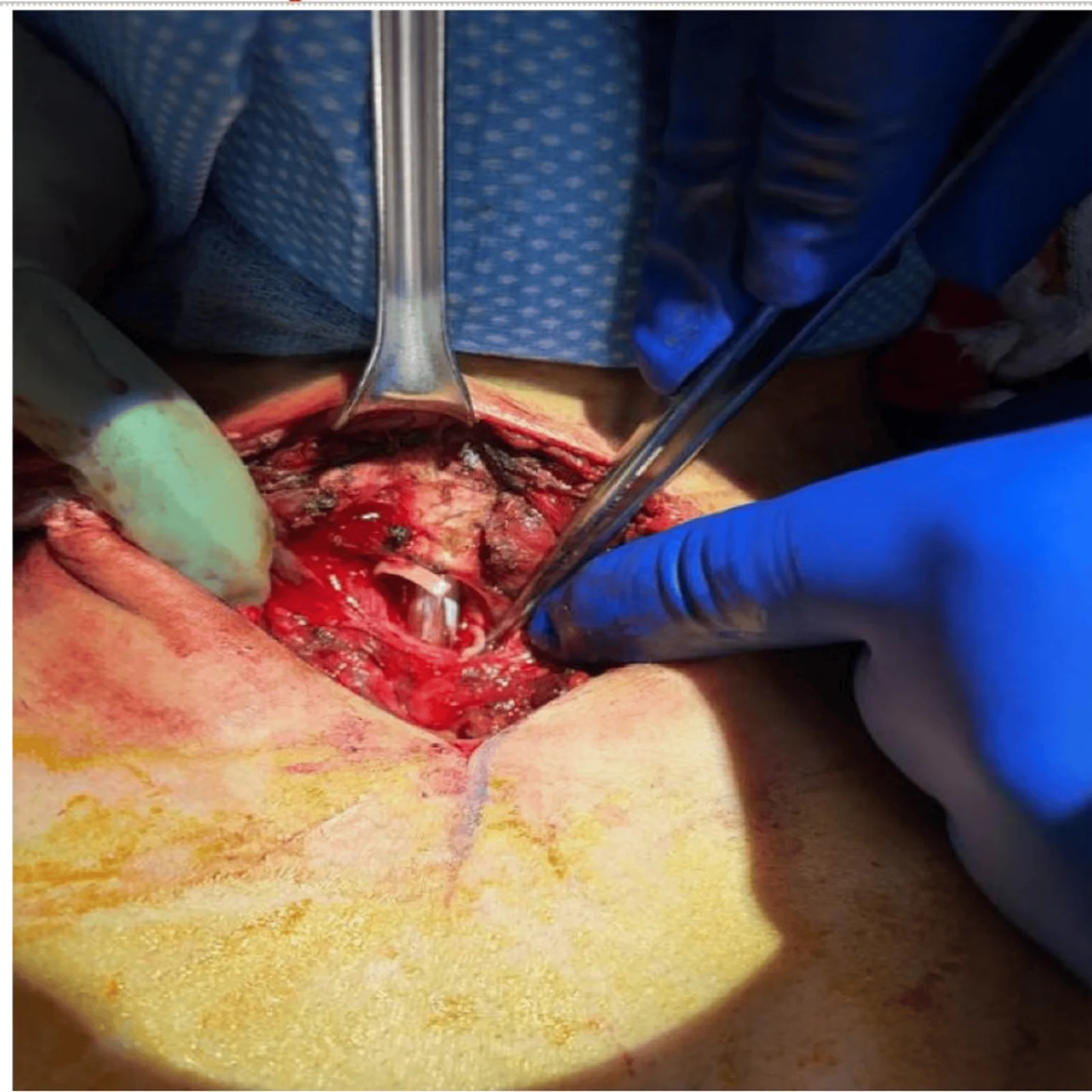
Visualization of the endotracheal tube noted through the anterior tracheal defect during intraoperative cervical exploration.

The patient was then taken emergently to the operating room. First, an esophagogastroduodenoscopy (EGD) was performed, and demonstrated no evidence of esophageal injury. Subsequent direct laryngoscopy revealed no injury to the larynx or cricoid cartilage. Both bronchoscopy and operative cervical exploration identified a near-complete laceration of the anterior tracheal wall involving the junction of the first and second tracheal rings (Figure 4). The posterior membranous trachea remained intact. The severely damaged segment, including the disrupted first and second tracheal rings, was excised. The proximal trachea was mobilized superiorly to the cricoid cartilage, and the distal trachea was mobilized inferiorly to the level of the carina with development of the bilateral paratracheal gutters, permitting tension-free approximation. Primary end-to-end tracheal anastomosis was performed circumferentially using interrupted 2-0 polydioxanone sutures with knots tied extraluminally. Cross-field ventilation was utilized during reconstruction, after which the oral endotracheal tube was advanced across the completed repair.

To protect the repair and secure the airway, a tracheostomy was created between the fourth and fifth tracheal rings, and a 6.0-mm distal extended-length cuffed tracheostomy tube was placed. Intraoperative tracheoscopy confirmed appropriate tube position approximately 1 cm proximal to the carina.

Postoperatively, the patient was admitted to the intensive care unit and maintained on mechanical ventilation for 24 hours. During this time, he was maintained on strict precautions to avoid excessive neck flexion or extension, and standard tracheostomy care was performed. The bilateral chest tubes were removed on postoperative day 2 after resolution of the pneumothoraces. On postoperative day 8, the patient returned to the operating room for bronchoscopy, tracheal dilation, and tracheostomy tube exchange under general anesthesia. Inspection at this time demonstrated satisfactory healing of the tracheal repair. Following evaluation by speech-language pathology, the patient was advanced to a regular diet with mildly thickened liquids and successfully evaluated for use of a Passy-Muir speaking valve (Passy-Muir, Inc., Irvine, California, USA). The patient progressed well overall with an uncomplicated postoperative course and was discharged on postoperative day 10 with scheduled outpatient follow-up with otolaryngology, cardiothoracic surgery, and speech-language pathology.

One month following the index operation, the patient underwent an outpatient follow-up evaluation with otolaryngology. He reported significant overall clinical improvement, including resolution of his dyspnea, stable phonation, and tolerance of oral intake without difficulty. He also had been successfully decannulated and was breathing comfortably without respiratory support. Given concern for possible early anastomotic narrowing, however, the patient underwent microdirect laryngoscopy and tracheal evaluation. Endoscopic evaluation demonstrated a well-healed tracheal anastomosis with a mildly stenotic segment at the prior repair site. The stenotic region was successfully managed with balloon dilation to approximately 16 mm using a controlled radial expansion balloon, followed by intralesional injection of triamcinolone (Kenalog 40) to reduce inflammatory scar formation. Repeat endoscopic evaluation demonstrated improved airway patency without evidence of dehiscence or airway instability. The patient tolerated the procedure well without any apparent complications. At the time of the patient’s most recent follow-up visit, he continues to do well with improved airway caliber and no evidence of respiratory syndromes and will continue outpatient surveillance for potential recurrent stenosis.

## Discussion

This case illustrates how misleading the initial presentation of a traumatic cervical tracheal injury can be, as well as how quickly the resultant complications may progress. Despite reassuring early findings, including stable vital signs and adequate oxygenation, there were subtle but key indicators of a more severe underlying injury. The high-energy mechanism of injury, a neck laceration violating the platysma, and mild voice changes all support a possible aerodigestive tract injury [1,2,4]. Although tracheobronchial injuries are relatively uncommon, delays in recognition or definitive airway establishment can lead to serious complications [1-5].

One of the more notable features in this case was the delayed appearance of significant subcutaneous emphysema accompanied by respiratory decline. The lack of subcutaneous emphysema early on should not be taken as evidence against airway injury, as temporary containment of air by surrounding tissues can obscure the diagnosis [1,2,4]. The rapid deterioration of our patient after airway manipulation and initiation of positive pressure ventilation highlights the need for ongoing reassessment and sustained clinical suspicion in cases of penetrating neck trauma [1,2,4,5].

Airway management in this setting presents particular challenges. In this case, initial endotracheal intubation resulted in tube misplacement through the tracheal defect, leading to poor ventilation, bilateral pneumothoraces, and a substantial air leak. This illustrates the risks associated with intubation when a tracheal injury is present and reinforces current recommendations that bronchoscopic guidance should be used whenever feasible to ensure appropriate tube placement distal to the injury [2,4-6]. Subsequent use of bronchoscopic guidance allowed for proper distal placement of the endotracheal tube and was followed by immediate improvement in ventilation, underscoring its importance in similar traumatic scenarios [2,4-6]. 

The later development of pneumomediastinum and bilateral pneumothoraces was likely due to preferential escape of air through the tracheal defect under positive pressure, a recognized complication of tracheobronchial injury [1,2,4]. Timely recognition and decompression were essential to stabilize the patient [2,4-6]. Definitive surgical management consisted of segmental tracheal resection with primary end-to-end anastomosis, performed in accordance with established principles emphasizing a tension-free repair and adequate tracheal mobilization [1,2,4,6]. Following primary repair of the defect, a tracheostomy was performed to secure the airway and minimize tension across the repair site [2,6]. The early identification of mild anastomotic narrowing, managed successfully with balloon dilation and steroid injection, highlights the importance of close postoperative monitoring to address complications before they progress clinically [1,2].

It is also important to recognize that the patient’s initial clinical deterioration and symptomatology raised concern for a possible allergic reaction, illustrating how evolving airway injuries can mimic other clinical diagnoses [1,4]. Overall, this case emphasizes the importance of maintaining diagnostic flexibility, coordinating care across specialties early, and using bronchoscopic guidance for airway management when tracheal injuries may be suspected [1,2,4-6].

## Conclusions

This case highlights the unpredictable presentation and rapid progression of traumatic tracheal injuries. The clinical course described above underscores how quickly airway compromise can develop, especially following positive pressure ventilation in the setting of an unrecognized tracheal defect. This case also reinforces the importance of careful airway management. Bronchoscopic guidance played a critical role in both the diagnosis and stabilization of our patient’s airway injury. Definitive surgical repair with tension-free primary anastomosis, combined with thoughtful postoperative management and surveillance, contributed to a favorable outcome despite the severity of the initial injury. Ultimately, maintaining a high clinical index of suspicion, performing serial clinical exams, and coordinating early multidisciplinary care are key to improving outcomes in patients with suspected tracheobronchial trauma.
